# SARS-CoV-2 in the ocular surface of COVID-19 patients

**DOI:** 10.1186/s40662-020-00189-0

**Published:** 2020-04-26

**Authors:** Hua-Tao Xie, Shi-Yun Jiang, Kang-Kang Xu, Xin Liu, Bing Xu, Lin Wang, Ming-Chang Zhang

**Affiliations:** 1 Department of Ophthalmology, Union Hospital, Tongji Medical College, Huazhong University of Science and Technology, Wuhan, 430022 China; 2 Department of Clinical Laboratory, Research Center for Tissue Engineering and Regenerative Medicine, Union Hospital, Huazhong University of Science and Technology, Wuhan, 430022 China

**Keywords:** SARS-CoV-2, COVID-19, Ocular surface

## Abstract

The 2019 novel coronavirus disease (COVID-19) caused by severe acute respiratory syndrome coronavirus 2 (SARS-CoV-2) has spread globally, while the routes of transmission of this virus are still controversial. We enrolled 33 patients, without any ocular manifestation, with their ocular surface swabs collected for virus detection. RNA was detected strong positive in samples of both eyes from two patients. Therefore, SARS-CoV-2 may exist in the normal ocular surface of COVID-19 patients, suggesting that this virus might be spread through conjunctival contact.

The type of pneumonia caused by the 2019 novel coronavirus disease (COVID-19) was first reported in Wuhan, China, in December 2019, followed by an outbreak of the country [1]. Analysis of lower respiratory tract samples revealed a novel coronavirus which was officially named severe acute respiratory syndrome coronavirus 2 (SARS-CoV-2) [2].

The main route of transmission is by respiratory droplets and direct contact. This virus were detected in the ocular surface of COVID-19 patients with conjunctivitis [3]. However, it is still unknown whether the virus can be detected on the ocular surface of COVID-19 patients without ocular manifestations, which may affect our precaution practices.

In this study, we retrospectively reviewed 33 consecutive COVID-19 patients without any ocular manifestation from February 12 to 28, 2020. The study was approved by Union Hospital ethics committee (approval number 20200009), and written informed consent was obtained from each enrolled patient. Diagnosis of COVID-19 was based on the Program for the Diagnosis and Treatment of Novel Coronavirus Infected Pneumonia (Trial Version 4) published by the National Health Commission of China [4]. All enrolled patients with COVID-19 were tested positive for SARS-CoV-2 by use of quantitative real time PCR (qRT-PCR) on samples from nasopharyngeal swab.

The ocular surface swabs from both eyes was taken within 7 days after the diagnosis of COVID-19, by putting a sterile cottonwool stick into the deep lower fornix of each patient’s eye after a single drop of topical anesthetic agent (1% amethocaine eye drops) was applied. All samples were processed simultaneously by detecting two target genes, including open reading frame 1ab (ORF1ab) and nucleocapsid protein (N) at the department of Clinical Laboratory of Union Hospital. Target 1 (ORF1ab): forward primer CCCTGTGGGTTTTACACTTAA; reverse primer ACGATTGTGCATCAGCTGA; and the probe 5′-FAM-CCGTCTGCGGTATGTGGAAAGGTTATGG-BHQ1–3′. Target 2 (N): forward primer GGGGAACTTCTCCTGCTAGAAT; reverse primer CAGACATTTTGCTCTCAAGCTG; and the probe 5′-FAM- TTGCTGCTGCTTGACAGATT-TAMRA-3′. The qRT-PCR assay was performed according to the manufacturer’s protocol (Shanghai Bio-Germ Medical Technology Co Ltd). Positive confirmatory were defined as those with a positive test result from either gene detection, and both positive was defined as strong positive.

Among the confirmed cases, the mean age was 57.6 (SD = 14.0) years and 22 (66.7%) were male. They were enrolled during the first (*n* = 5, 15.2%), second (*n* = 17, 51.5%), third (*n* = 8, 24.2.3%) and fourth (*n* = 3, 9.1%) weeks of their diseases. No ophthalmic complication was reported after the swab. SARS-CoV-2 RNA was detected strong positive in samples of both eyes from two patients. One patient was a 90-year-old female. She came to the hospital because of fever, headache and dyspnea. She died of multiple organ failure 8 days after onset of the disease. Her ocular surface samples were collected 5 days from the onset. The other patient was a 48-year-old male. He recovered after 3 weeks’ supporting treatment. The ocular samples were taken 4 days from the onset of fever and cough.

SARS-CoV-2 was tested strong positive in both eyes from 2 of 33 patients by detecting two different target genes. The low positive rate may be caused by multiple factors. Firstly, 74.8% samples were collected more than 1 week after the onset of the disease. This phenomenon is similar in patients with SARS that the secretion of virus in tears occurs only during the early phase of the disease [5]. Secondly, ocular surface swab samples collected few exfoliated cells which had a low chance of detecting the virus. Finally, the lack of sensitivity of RT-PCR kits may also lead to a low positive rate, which was also an notorious problem in the detection of SARS-CoV [6].

Xia et al. reported that SARS-CoV-2 existed only in one patient’s tear and conjunctival secretions with conjunctivitis and speculated that the tear and conjunctival secretions of patients without conjunctivitis were not an infectious source for SARS-CoV-2 [3]. Our present study demonstrated that the SARS-CoV-2 RNA was detected from the normal ocular surface of COVID-19 patients. The disparate phenomenon might result from the different target genes used between the two studies. Yu Jun et al. obtained negative results when analyzing tear samples collected from Schirmer’s test strip for SARS-CoV-2 by RT-PCR and viral isolation [7]. The Schirmer’s test strips collect few conjunctival exfoliated cells and secretions which are also potential infectious sources of ocular surface especially when patients rub their eyes. The negative results cannot suggest a low risk of ocular spread.

According to the results of this study, virus was detected positive in tear and conjunctival secretions in patients without conjunctivitis. The most commonly used non-contact tonometer for ophthalmologic examination sprays a gas during examination, which produces a large amount of aerosol in the local area. Therefore, the examination should be operated in ventilated place and air disinfection should be employed. The ophthalmologic staff must pay more attention to eradicate cross infection during examination of ultrasound biomicroscopy, corneal confocal microscope, and others.

In conclusion, our study indicates that SARS-CoV-2 might spread from normal conjunctiva of COVID-19 patients, which has enriched our understanding of the transmission routes of the virus.

## Data Availability

The datasets used and/or analysed during the current study are available from the corresponding author on reasonable request.

## Abbreviations

SARS-CoV-2: Severe acute respiratory syndrome coronavirus 2
COVID-19: 2019 novel coronavirus disease
qRT-PCR: Quantitative real time PCR
